# Editorial: Platelet Function in COVID-19

**DOI:** 10.3389/fcvm.2022.912472

**Published:** 2022-05-26

**Authors:** Annika Lundström, Per Sandén, Ioannis Mitroulis, Paola E. J. van der Meijden

**Affiliations:** ^1^Department of Clinical Sciences, Danderyd Hospital, Karolinska Institutet (KI), Stockholm, Sweden; ^2^Laboratory of Molecular Hematology, Department of Medicine, Democritus University of Thrace, Alexandroupolis, Greece; ^3^Department of Biochemistry, Cardiovascular Research Institute Maastricht, Maastricht University, Maastricht, Netherlands

**Keywords:** platelet, COVID-19, immunothrombosis, platelet activation and reactivity, antithrombotic therapy

More than 2 years into the SARS-CoV-2 pandemic, it is clear that platelets have a role in COVID-19 pathogenesis and likely increase the risk of thrombosis, even though findings are partly contradictory and exact mechanisms remain obscure. The present Research Topic was launched to clarify aspects of platelet function in COVID-19 and to identify future research areas. We are happy to present five papers: a meta-analysis of the association between COVID-19 severity and platelet count respectively routine coagulation tests (Len et al.), a review of molecular and cellular thrombotic mechanisms in COVID-19 (Mizurini et al.) and three original papers from one clinical study reporting different aspects of platelet function and their temporal evolution in hospital-treated COVID-19 patients (Schrottmaier et al., Schrottmaier et al., Ercan et al.).

Low platelet counts are common in viral disease; generally mild to moderate reductions are seen in COVID-19. Early studies reported increased risk of severe or lethal COVID-19 for patients with thrombocytopenia (1, 2), similar to findings for other viral diseases (3). In one of the larger meta-analyses to date, Len et al. confirm a lower mean platelet count early in the course of COVID-19 for patients developing severe disease as compared to those with mild to moderate forms. In line with the concept of COVID-19 associated coagulopathy (CAC) (4), fibrinogen and D-dimer were higher and APT and PT longer in severe cases. A number of explanations for reduced platelet counts in COVID-19 have been proposed, including platelet trapping in microthrombi/thrombi (5). Len et al. discuss possible COVID-19-induced effects on thrombopoiesis including megakaryocyte infection by SARS-CoV-2 and the role of tissue megakaryocytes. Barrett et al. recently observed virions in bone-marrow megakaryocytes of one COVID-19 autopsy case, indicating that megakaryocytes can be infected by SARS-CoV-2 *in vivo* (6). It remains to be established if this a common phenomenon and how circulating platelets are affected. After hospital admission platelet counts often increase in COVID-19, peaking on day 8 in a large cohort (6). Interestingly, a recent study showed that increasing platelet counts over time (more than 10 x 10^9^/l/day) was associated with reduced risk of death and thrombosis compared to stable or decreasing values (7). Len et al. note that autopsy studies show increased numbers of pulmonary megakaryocytes and presence of tissue megakaryocytes in hearts and brains of deceased COVID-19 patients (for references see Len et al.). Megakaryocyte migration to tissues and possible tissue-based platelet production may affect circulating platelet counts and platelet phenotype. Notably, animal models show immune-modulating functions in pulmonary megakaryocytes (8, 9). Factors determining platelet counts, megakaryocyte migration to tissues and possible tissue-based platelet production in COVID-19 are areas that warrant further research.

Immunothrombosis is considered a major factor in CAC and the thrombogenicity of COVID-19. It involves endothelial dysfunction, activation of innate immune cells, NETosis, platelet and complement activation as well as the coagulation system. Mizurini et al. provide a comprehensive review of cellular responses, cell-cell interactions and molecular pathways in COVID-19 immunothrombosis. Platelets have a role in many of these mechanisms, not least *via* their immunological functions and extensive interactions with other cell types. The review summarizes the evidence for elevated basal platelet activation, degranulation, response to agonists and interactions with leukocytes in COVID-19. It has been debated if platelets express the main SARS-CoV-2 receptor angiotensin-converting enzyme 2 (ACE2) and/or take up SARS-CoV-2 (10). A recent study verified the presence of fragmented, likely digested SARS-CoV-2 RNA in platelets from COVID-19 patients (11). RNA sequencing and Western blot showed increased expression of pathways for programmed cell death. *In vitro*, several mechanisms for platelet uptake of SARS-CoV-2 were observed. These were partly ACE2 dependent but virus could also be taken up independent of ACE2, in particular when associated with microvesicles. Thus platelets contribute to virus elimination by mechanisms that appear partly SARS-CoV-2 specific and may alter platelet function.

The AVOVACT trial (NTC04351724) investigated several aspects of platelet function depending on disease severity in hospitalized COVID-19 patients in Vienna. In agreement with other studies, Schrottmaier et al. found elevated basal platelet activation at admission. However, platelets from patients with lethal COVID-19 in contrast to survivors displayed decreasing GPIIbIIIa activation over time and also decreasing response to agonist-induced activation. Levels of platelet GPIIb measured by proteomics also decreased over time in lethal COVID-19 (Ercan et al.). Based on other measured biomarkers, the authors question if platelet exhaustion fully explains the result.

The association of antiplatelet and anticoagulant treatment with outcome was investigated in a larger cohort of AVOVACT in Schrottmaier et al. Anticoagulant treatment was associated with improved survival while antiaggregant treatment had no effect. The study was observational but a recent large randomized trial on the effect of aspirin in COVID-19 gave a similar result (12). Complementary investigations in Schrottmaier et al. showed normal spreading of COVID-19 platelets on fibrinogen and in autopsies only limited platelet trapping in pulmonary thrombi of small or intermediate size. Together the results suggest that platelet activation in COVID-19 does not primarily result in enhanced platelet aggregation *in vivo*.

In summary, the papers of the Research Topic expand and nuance findings from previous studies on platelet function in COVID-19. At least fatal cases appear characterized by a gradual loss or suppression of platelet responsiveness. Larger studies are needed which should consider symptom duration at the time of testing, patient selection and methodological difficulties. Several aspects of platelet function in COVID-19 are still unresolved, e g the role of plasma-based platelet activating factors such as IgG subtypes, activation of ITAM-receptors, shifts in platelet phenotype (pro-coagulant vs. pro-aggregatory) and alternative antiplatelet treatments (13–15). Clarifying these issues may be valuable not only for COVID-19 but also for other infectious diseases and possibly thrombotic diseases in general.

## Author Contributions

AL drafted and revised the editorial. PS, IM, and PM reviewed and edited the manuscript. All authors contributed to the article and approved the submitted version.

## Conflict of Interest

The authors declare that the research was conducted in the absence of any commercial or financial relationships that could be construed as a potential conflict of interest.

## Publisher's Note

All claims expressed in this article are solely those of the authors and do not necessarily represent those of their affiliated organizations, or those of the publisher, the editors and the reviewers. Any product that may be evaluated in this article, or claim that may be made by its manufacturer, is not guaranteed or endorsed by the publisher.
